# Hospital Meetings, &c.

**Published:** 1900-11-03

**Authors:** 


### HOSPITAL MEETINGS, &C.

### THE HOME HOSPITALS ASSOCIATION.

The Committee of the Home Hospitals Association for Paying Patients, Fitzroy Square, at a meeting last week (Thursday, October 25th) considered a suggestion put forward by Mr. Bland Sutton to bring the institution up to date by the provision of operating theatres, sterilising rooms, and a lift. A proposal was also mentioned to rebuild the premises, taking in two or three adjoining houses, and by this means providing room for an additional branch which should provide beds for a poorer class of paying patients. After considerable discussion a decision was come to not to embark on the larger scheme, though the desirability of such provision was recognised; and a committee consisting of Mr. Cox, Mr. Bryant, Sir Henry Burdett, and the Hon. Sec. (Mr. Alward Hind) was appointed to go into the question considering it from the structural, financial, and general aspects of the matter.

### THE FREE HOME FOR THE DYING.

The dedication of the new and permanent home of "The Free Home for the Dying," 29 North Side, Clapham Common, took place on Friday afternoon last. The ceremony was performed by the Right Rev. the Bishop of Southwark. The new building was formerly the residence of the late Sir Charles Barry, and is one of the few fine old houses facing the Common still remaining. The home has undergone considerable alteration at the hands of the well-known architect, Mr. A. E. Street, who has himself made a donation of £100 towards the building fund. The purchase of the freehold and the cost of the alterations and repairs have involved an outlay of more than £10,000. Of this amount a sum of £3,000 has still to be collected. The Bishop, in the course of his address, stated that a munificent donation of £1,000 had been given that day to permanently endow one of the beds. The new building contains twenty-five beds. Of these twelve are in one large and well-proportioned room—the women's ward. The men's ward contains eight beds, and there are several beds in separate rooms. The home is absolutely free, without payment of any kind, and without distinction of creed. All that is necessary is a doctor's certificate that the patient is in the last stages of disease. The work, which will now be on a much larger scale, is carried on by the Sisters of St. Margarets, East Grinstead, in whose hands it has already been for some years.

